# Tuning Deep Learning for Predicting Aluminum Prices Under Different Sampling: Bayesian Optimization Versus Random Search

**DOI:** 10.3390/e28020145

**Published:** 2026-01-28

**Authors:** Alicia Estefania Antonio Figueroa, Salim Lahmiri

**Affiliations:** Department of Supply Chain and Business Technology Management, John Molson School of Business, Concordia University, Montreal, QC H3H 0A1, Canada; alicia.antoniofigueroa@mail.concordia.ca

**Keywords:** deep learning, LSTM, deep feedforward neural networks, support vector regression, Bayesian optimization, random search, aluminum price, forecasting

## Abstract

This work implements deep learning models to capture non-linear and complex data behavior in aluminum price data. Deep learning models include the long short-term memory (LSTM) and deep feedforward neural networks (FFNN). The support vector regression (SVR) is employed as a base model for comparison. Each predictive model is tuned by using two different optimization methods: Bayesian optimization (BO) and random search (RS). All models are tested on daily, weekly, and monthly data. Three performance metrics are used to evaluate each forecasting model: the root mean squared error (RMSE), mean absolute error (MAE), and the coefficient of determination (R^2^). The experimental results show that the LSTM-BO is the best-performing model across the time horizons (daily, weekly, and monthly). By consistently achieving the lowest RMSE, MAE, and highest R^2^, the LSTM-BO outperformed all the other models, including SVR-BO, FFNN-BO, LSTM-RS, SVR-RS, and FFNN-RS. In addition, predictive models utilizing BO regularly outperformed those using RS. In summary, LSTM-BO is highly beneficial for aluminum spot price forecasting.

## 1. Introduction

Aluminum is essential for modern industrial applications, reshaping sectors from transportation to urban development with its unique properties. The aluminum market was valued at USD 147.2 billion and is projected to increase to USD 189.8 billion by 2026, with an annual growth rate of 3.2% from 2019 to 2026. This substantial growth reinforces the importance of intensifying research on this metal market [1]. For instance, the demand for lightweight automobiles in the automotive industry promotes the use of aluminum to replace traditional steel structures [1]. This trend leads to the growth of aluminum demand, and its versatility offers a wide range of applications in various industries. As industries increasingly adopt sustainable practices, procurement managers have the opportunity to adapt their strategies to include green sourcing, as it requires 95% less energy compared to the production of primary aluminum [2]. For instance, the authors in [1] emphasized the need to replace metals that have reached their performance limits for aluminum.

In this regard, various works focused on the analysis and modeling of aluminum market prices. For instance, the authors in [1] used variational mode decomposition (VMD) and LSTM to predict the aluminum market price. The model outperformed auto-regressive moving average (ARIMA) and feedforward neural networks. In ref. [3], variational mode decomposition was employed to decompose the original price series into several components. Then, an LSTM network was used to predict each component. Finally, the forecasting results of each component were combined to formulate the final predicted output for the original price series. In ref. [4], the authors examined how the exchange rates of commodity-exporting countries, especially Chile and New Zealand, can predict the prices of aluminum for spot and future contracts every quarter. They used an auto-regressive (AR) process as a predictive modeling approach to effectively select and shrink the coefficients of exchange rates that were less relevant in forecasting aluminum prices. PCA reduces the dimensionality of the data, focusing on the first principal component of the exchange rates to capture the most significant variance and use it for forecasting. It was concluded that the exchange rates of some commodity exporter countries can forecast the spot price and aluminum future contracts. In ref. [5], the authors combined the generalized autoregressive conditional heteroskedasticity (GARCH) model with LSTM, Bi-LSTM, and standard artificial neural networks. It was concluded that due to the different complexities in the prices, using the long-memory properties of models like Bi-LSTM or LSTM will achieve good predictive results if we have significant non-linear, highly complex price dynamics.

The sequence dependence complexity among the input variables of the stock prices of aluminum was examined in [6] by using LSTM. The study successfully provides a forecasted result for the next four months, showing an upward trend for a few weeks and then a decrease in the trend. The predicted values were tested against the actual ones for two weeks, showing an alignment of both datasets. The authors concluded that LSTM successfully outperformed models in their literature. A hybrid model was presented in [7] to predict zinc, aluminum, copper, and gold prices. First, price series were decomposed by using improved complete ensemble empirical mode decomposition with adaptive noise. Then, the resulting subseries were predicted by a non-linear autoregressive model, backpropagation neural networks, LSTM, recurrent neural networks, and ARIMA models. The final residual prediction result is obtained by screening and adding the best findings from each subsequence. Lastly, the Prophet’s prediction results and the residual prediction results are added to obtain the final metal price forecast value. For aluminum, the correction effect of LSTM is better than the others in the model selection. In ref. [8], the authors used complete ensemble empirical mode decomposition with adaptive noise to break down the original price sequence. Then, VMD was used to further decompose the most complex subsequence. To improve the efficiency of VMD, particle swarm optimization was employed to fine-tune its parameters. The LSTM was utilized to predict each subsequence. The final prediction was obtained by summing the forecasts from each subsequence. The proposed model outperformed other benchmark models, proving its robustness, but was limited by its computational time.

In ref. [9], the authors predicted aluminum and copper prices by comparing several layers of neural networks with a layer of LSTM, considering a 30-day delay. Their best model considers a three-layer neural network, a layer of LSTM, and a 30-day delay to most accurately predict aluminum prices. Additionally, their study examined the relationship between aluminum and copper prices using Pearson’s correlation coefficient, to contribute to the importance of metal commodity substitutes in developed countries to mitigate metal supply disruptions. The authors in [10] proposed a price prediction model for copper, aluminum, and zinc datasets. The variational mode decomposition (VMD) and wavelet packet transform were employed to decompose the original series. Then, ARIMA and extreme learning machines (ELM) were employed to forecast, respectively, the components obtained by VMD and wavelet packet transform. The prediction outputs of each component were accumulated to obtain the initial forecasting results and errors. Next, a wavelet packet was used to further decompose the error sequence, and the error subsequence was predicted by ARIMA and ELM optimized by an improved sparrow search algorithm to obtain the error prediction results. Lastly, the error prediction results were used to correct the initial forecasting results, and the final forecasting results of non-ferrous metal prices were obtained. For the aluminum price, there were improvements shown in the performance metrics with the use of the second stage decomposition using wavelet packet decomposition. This novel model proved significant for predicting price fluctuation, with its reduced percentages in performance metrics.

In ref. [11], hybrid models were designed to predict prices of aluminum, aluminum alloy, copper, lead, tin, gold, silver, platinum, and palladium. Using hybrid models such as support vector regression (SVR) tuned by grey wolf optimization, LSTM-SVR, LSTM-CNN (convolutional neural networks), and LSTM-GRU (gated recurrent units), their findings indicate that the LSTM-GRU model is the most effective for predicting aluminum prices, demonstrating high accuracy. The VMD technique was adopted in [12] to decompose price series, and LSTM was optimized by using the sparrow search algorithm optimization (SSA) to forecast each decomposition unit. Finally, the prediction results of each decomposition unit were summed to obtain the final forecast. The validation results show that the VMD-SSA-LSTM model outperformed other comparative models in terms of forecasting accuracy, including LSTM, SSA-LSTM, and VMD-LSTM.

The main purpose of this study is to forecast the aluminum spot price using deep learning, namely the LSTM system. The LSTM is chosen because it has been shown to be successful in previous works [5,6,7,8,9,11,12]. The main contributions to the literature are as follows. First, two powerful machine learning models are used as baseline models for comparison against the LSTM: the deep feedforward neural networks (DFFNN) system, which is the basic deep learning model with several hidden layers, and SVR, which is a powerful statistical learning model based on structural risk minimization theory. Second, to fine-tune the predictive models, Bayesian optimization (BO) and random search (RS) algorithms are implemented. Then, the performance of each predictive model is assessed under each optimization algorithm. Third, three different forecasting time horizons are considered, including daily, weekly, and monthly horizons. Hence, manufacturing companies in the aluminum industry, which are known to be subject to price sensitivity, can be equipped with the best combination of predictive models and optimization techniques to develop a general strategy for each forecasting time horizon.

The rest of the paper is organized as follows: Section 2 presents the machine learning models, optimization methods, and performance measures. Section 3 describes the data and provides the results. Finally, discussion and conclusion are provided in Section 4.

## 2. Methods

**Figure 1:**
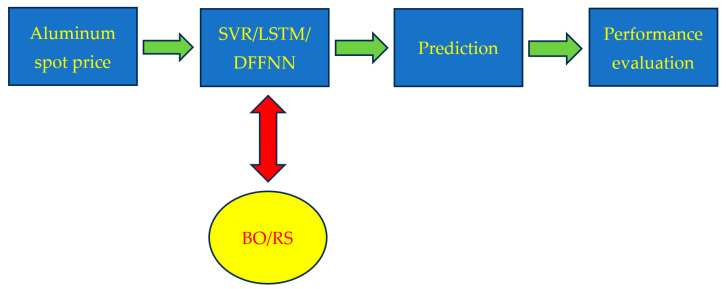
Flowchart of the experiments.

The spot price of aluminum was modeled and predicted by using SVR, LSTM, and DFFNN. Two types of experiments were conducted. In the first one, each predictive model was optimized by using Bayesian optimization. In the second experiment, each predictive model was tuned by using random search. Finally, the performance of each model under each optimization technique was assessed based on performance metrics. Figure 1 shows the flowchart used to summarize the experiments. The SVR, LSTM, DFFNN, Bayesian optimization (BO), random search (RS), and performance measures are described next.

### 2.1. Support Vector Regression

The support vector regression (SVR) [13] is a non-linear statistical machine learning model that minimizes structural risk. The SVR approximates the function *f*(*x*) used to represent the output (prediction) as follows:

(1) $$f\left(x\right)=\sum_{k=1}^{N}\alpha_{k}K\left(x,x^{\prime }\right)+b$$

where x and x′ stand for two data, *α* is a Lagrange multiplier, *b* is a parameter, and *K* is a kernel function. Indeed, the structure utilizes a kernel function expressed as K(x,x′) used to transform the input data into a higher-dimensional space. The most used kernel functions are polynomial kernels, sigmoid kernels, and radial basis functions, given below:

Polynomial kernel:

(2) $$K\left(x,x^{\prime }\right)={\left(\gamma \langle x,x^{\prime }\rangle +b\right)}^{d}$$

Sigmoid kernel:

(3) $$K\left(x,x^{\prime }\right)=\left(tanh\left(\gamma \langle x,x^{\prime }\rangle \right)+b\right)$$

Radial basis function:

(4) $$K\left(x,x^{\prime }\right)=exp\left(-\gamma {\left\|x-x^{\prime }\right\|}^{2}\right)$$

where *d* and *γ* are kernel parameters. In the SVR framework, most of the Lagrangian multipliers, α values, turn out to be zero. This means that only a subset of the training data (the support vectors) is used in the decision function, making the model both efficient and effective, especially when dealing with large datasets.

### 2.2. Deep Feedforward Neural Networks

The feedforward neural network (FFNN) [14] is a multilayer perceptron composed of an input layer, a hidden layer, and an output layer. The neurons of end-to-end layers are connected by weights. The signal passes only from the input layer to the first hidden layer, then to the output layer. The output (prediction) of the network is given by

(5) $$y_{i}=f\left(\sum_{j=1}^{n}w_{ij}x_{ij}+w_{i0}\right)$$

where *w_ij_* is the weight of the link from neuron *j* of the previous layer to the current neuron *i*, *x_ij_* is the corresponding signal, *w_i_*_0_ is the inherent threshold of neuron *i*, and *f* is an activation (transfer) function. At iteration *i*, the weights of the network are adapted as follows:

(6) $$∆w_{i}=-\gamma_{i}G_{i}$$

where *γ* is the learning rate, and *G* is the gradient. In the feedforward neural network, the neurons in the first layer are connected to all the neurons in the next layer, and the data are propagated in the entire network without feedback or a loop process. The major difference between Deep-FFNN (DFFNN) and a shallow feedforward neural network (FFNN) is the number of hidden layers used to determine the depth of the network. Specifically, the DFFNN has more than one hidden layer, whilst the shallow FFNN has only one hidden layer.

### 2.3. Long Short-Term Memory

LSTM [15] is a type of deep recurrent neural network (RNN), known for its effectiveness in predicting time series data, and recognized for its ability to process data sequences while retaining information for extended periods [16]. This specialized neural network includes memory cells that allow it to process data with periodic patterns and handle long-term dependencies effectively. As is common within neural network models, LSTM works under an input layer, a hidden layer, and an output layer. In the hidden layer, LSTM processed a chain of repeating cells of a neural network. Each memory cell in LSTM has three types of gates: forget gate, input gate, and output gate. These gates control the flow of information, helping the network to remember important information and discard the unnecessary ones. The design of LSTM allows it to avoid the problems of vanishing and exploding gradients, making it particularly good at learning from long sequences of data. This selective memory is crucial for effectively modeling the complexities and non-linear characteristics of volatile data sequences.

The cell state mechanism is the core of its design, composed of a symphony of gates: the input gate $\left(i_{t}\right)$, the forget gate $\left(f_{t}\right)$, and the output gate $\left(o_{t}\right)$, which together manage the flow of information. Weights and biases (W and b) correspond to the weight matrices and bias parameters within the network. The forget gate is given by

(7) $$f_{t}=\sigma \left(W_{f}\left[h_{t-1},X_{t}\right]+b_{f}\right)$$

Here, the forget gate $\left(f_{t}\right)$ regulates the flow of information from the current input and the previous hidden state $\left(h_{t-1}\right)$ by applying a sigmoid activation function, selectively keeps or discards previous information, guaranteeing that the cell state $\left(C_{t}\right)$ is a dynamic representation of learned data over time. The following step is to use the input gate $\left(i_{t}\right)$ to manage the patterns of data flowing from the current input and the previous hidden states. Cell State ($C_{t}$) updates with contributions from the input gate, forget gate, and previous cell state ($C_{t-1}$), and the transformed input using the tanh function.

(8) $$i_{t}=\sigma \left(W_{i}\left[h_{t-1},X_{t}\right]+b_{i}\right)$$

(9) $$C_{t}=f_{t}⊙C_{t-1}+i_{t}⊙\tanh\left(W_{c}\cdot \left[h_{t-1},x_{t}\right]+b_{c}\right)$$

Finally, the output gate ($O_{t}$) determines the necessary information for the activation function of the output (prediction) value ($h_{t}$).

(10) $$O_{t}=\sigma \left(W_{o}\left[h_{t-1},X_{t}\right]+b_{o}\right)$$

(11) $$h_{t}=O_{t}\tanh\left(C_{t}\right)$$

### 2.4. Bayesian Optimization

Bayesian optimization [17] utilizes prior information from previous parameter sets to determine the next set to be evaluated. This method can learn and select hyperparameter sets based on their distributions by defining fitness scores from previous iterations that require fewer function evaluations than other classical optimization methods [18]. This search provides higher efficiency with fewer iterations and the ability to accurately find the optimal hyperparameter solution [19]. BO has two main parts. The first part is the probabilistic surrogate model that replicates the behavior of the expensive objective function. The second part is the scalarization of the probabilistic model using an acquisition function to predict the next point of evaluation [20].

Based on several iterations, BO builds a probabilistic model of the function being optimized and selects the next set of hyperparameters to assess based on the current best estimated one from the function behavior, resulting in the best optimal set of hyperparameters for a machine learning model. By repeatedly updating the probabilistic model, the BO evaluates the space of hyperparameters and converges to the optimum set with a minimum number of function evaluations [21]. Thus, to undertake this, the BO uses a stochastic surrogate model to simulate an expensive objective function based on a finite number of function observations.

The main objective function f(x) of hyperparameter optimization is the performance of artificial intelligence models. Solving this function yields the values that maximize this performance metric [22]. The process of searching for the optimal hyperparameters is performed through a Gaussian Process (GP), using this type of stochastic model to guide the search for the optimal hyperparameters. The function *f*(*x*) follows a GP:

(12) $$f(x)\sim GP(m\left(x\right),k\left(x,x^{\prime }\right))$$

GP is known for being a powerful non-parametric method well-suited for approximating unknown functions, especially when dealing with black-box functions where explicit forms are unknown [17]. The strength of GPs lies in their ability to provide a probabilistic forecast of function values, leveraging the covariance between points to predict outcomes for new, unseen data.

To select the next set of hyperparameters to evaluate, BO relies on acquisition functions (AF). The acquisition function calculates the expected utility of evaluating the objective function at a new point [23]. The main goal of this function is to try new regions of the hyperparameter space and focus on regions that are known to work well. BO is centered on building the unknown objective function as a probabilistic model. In general, these acquisition functions depend on the previous observations, as well as the GP hyperparameters. Commonly used acquisition functions include expected improvement (EI), probability of improvement (PI), and upper confidence bound (UCB). In this study, the EI function is adopted. For instance, the EI function is used to measure the expected degree of improvement over the current best observation at a point *x*, considering both the mean and variance in the predictive distribution. Therefore, the local optimal solution may be the current ideal value point, and the algorithm will identify the best value point in other domain positions. The EI function makes it more difficult to reach the local optimum solution. The EI function is given by

(13) $$EI=\int_{\infty }^{\infty }If\;\left(I\right)dI=\int_{I=0}^{I=\infty }1\frac{1}{\sqrt{2\pi \sigma (X)}}\exp\left(-\frac{\mu \left(x\right)-f\;\left(x^{+}\right)-I{)}^{2}}{2\sigma^{2}\left(x\right)}\right)dI=\sigma \left(x\right)\left[Z\emptyset \left(Z\right)+\varphi (Z)\right]$$

where I denotes the expectation of improvement in EI. It is worth noting that the choice of the expected improvement as the acquisition function is justified by its ability to balance exploration and exploitation, its computational efficiency, and its strong empirical performance as it guides the search by maximizing the expected gain over the current best point.

### 2.5. Random Search

In random search [24,25,26], the objective is often to find the set of hyperparameters that minimizes a specific metric. In our research, we selected to minimize the validation loss, which provides a direct measure of a model’s ability to generalize beyond the training data. By using this optimization function, the search aims to optimize the model’s performance on unseen data. Under the RS algorithm, the validation loss function is given by

(14) $${Min}_{\theta \in \Theta }\;f\;(\theta )$$

where Θ is the feasible region and f: Θ → R is the objective function. The feasible region Θ consists of all the possible designs of a system under consideration, and *f*(θ) denotes the expected performance of that system under the design θ ∈ Θ. Random search methods involve sampling points from the feasible region Θ of the underlying optimization problem using a sampling strategy and evaluating the performance of the objective function f at the chosen points. Then the method proceeds to update the sampling strategy based on the current feasible points that have been sampled and the associated objective function values, before proceeding to the next iteration. Consequently, a large class of optimization techniques known as random search methods can be used to tackle deterministic and stochastic optimization problems with discrete or continuous decision parameters (or both). As a specific instance of stochastic optimization, simulation optimization involves considerable noise because the necessary objective function values *f*(θ) are determined by computer simulation. Thus, in summary, the RS algorithm operates by evaluating the objective function *f* over several randomly selected hyperparameter sets from a predefined hyperparameter space Θ.

### 2.6. Performance Measures

In this study, three common evaluation metrics are used to assess the performance of each predictive model under each optimization technique. They are RMSE, MAE, and R-squared (R^2^). They are chosen in our study as they are common in applications of machine learning in time series forecasting and widely used in the literature [1,2,3,4,5,6,7,8,9,10,11,12]. The RMSE represents the residuals’ standard deviation, or the average difference between the predicted values and the actual ones. The RMSE is expressed as follows:

(15) $$RMSE=\sqrt{\frac{1}{n}\sum_{i=1}^{n}{\left(y_{i}-{\hat{y}}_{i}\right)}^{2}}$$

where $y_{i}$ represents the actual value of the target variable for the ith observation, ${\hat{y}}_{i}$ is the predicted value of the target variable for the ith observation, and n is the total number of observations. It is worth mentioning that the universal measure, Euclidean distance, which refers to RMSE without the square root, could be used.

MAE is a statistical measure of the average of the absolute errors between the predicted values generated by the model and the actual values in the dataset, without considering the direction of the errors. The MAE is also referenced in the literature as the Manhattan distance. It is robust to outliers because it does not square the errors; the smaller the MAE, the better the model’s performance, and the closer it is to 0, the more accurate the model is. It is expressed as follows:

(16) $$MAE=\frac{\sum_{i=1}^{n}\left|y_{i}-{\hat{y}}_{i}\right|}{N}$$

where $\left|y_{i}-{\hat{y}}_{i}\right|$ denotes the absolute value error for each individual prediction.

Finally, the R^2^ is used to measure how well a model fits the data. For instance, quantifies how much of the variation in the data is explained by the model. This statistic is calculated based on the sum of squares of residuals (SSR) and the total sum of squares (SST). Specifically, R^2^ is computed as follows:

R^2^ = 1 − (SSR/SST) (17)

where SSR is the sum of the squares of the differences between the actual $y_{i}$ and the predicted ${\hat{y}}_{i}$ by the model. It measures the amount of variance in the dependent variable that the model does not explain. It is given by

(18) $$\mathrm{SSR}=\sum_{i=1}^{n}{\left(y_{i}-\hat{y}\right)}^{2}$$

SST is the total sum of the squares of the differences between the actuals $y_{i}$ and the actual mean values $\bar{y}$. It measures the total variance in the dependent variable as follows:

(19) $$\mathrm{SSR}\;=\;\sum_{i=1}^{n}{\left(y_{i}-\bar{y}\right)}^{2}$$

The R^2^ provides a quick view of the model’s effectiveness in explaining the variability of the data.

## 3. Data and Results

**Figure 2:**
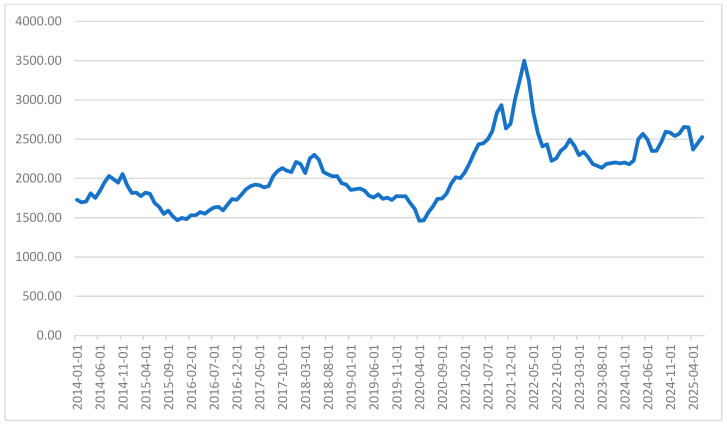
Monthly spot price of aluminum in US dollars.

The dataset selected has a span from 16 June 2014 to 22 February 2024. Aluminum spot prices from the London Metal Exchange (LME) [27] are one of the largest markets for trading industrial metals. The predictive models (SVR, DFFNN, and LSTM) are assessed under standard partition rules, allocating 80% of the data for training and 20% for testing the predicted values against the actual observations. Figure 2 exhibits the monthly evolution of the aluminum spot price.

**Figure 3:**
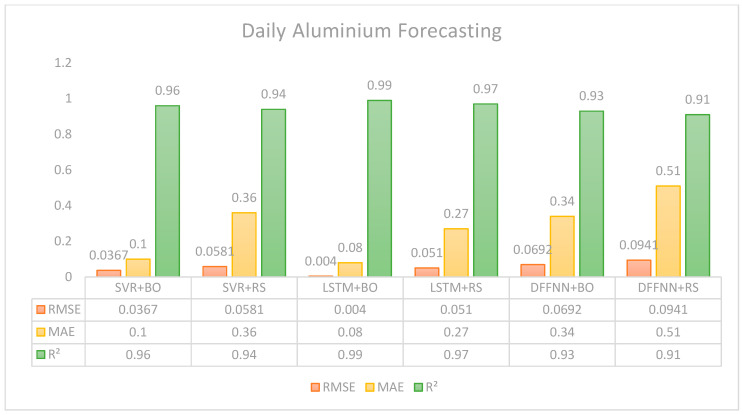
Forecasting results based on daily data.

Regarding daily forecasting results shown in Figure 3, the statistical measures from LSTM with Bayesian optimization outperform its random search counterpart, offering substantially lower errors and a well-fitted R^2^. This model shows the best overall performance across all configurations, suggesting high efficiency in capturing temporal dependencies in the data. On the other hand, SVR performs better with Bayesian optimization, showing lower error rates, RMSE 0.0367, MAE 0.1, and 0.96 R^2^. Thus, it indicates a better fit and prediction accuracy than with random search, under which it achieved RMSE 0.0581 and MAE 0.36. In terms of errors, we can suggest that the prediction of this model is still considered a good fit for the data. However, R^2^ suggests that the model’s ability to explain the predicted values is 0.94 against 0.96 as observed from the Bayesian hyperparameter tuning. Lastly, DFFNN shows the least favorable outcomes among the three models. Particularly when using random search, the DFFNN model yields the highest RMSE 0.0941 and MAE 0.51 errors, and the lowest R^2^ 0.91 of the daily forecasting. Even though the Bayesian optimization helped improve performance, DFFNN still underperforms the other models as it shows higher RMSE.

In summary, Figure 3 indicates that all predictive models perform better under BO than under the RS optimization algorithm. In addition, LSTM-BO is overall the best predictive system to forecast the aluminum daily spot price.

**Figure 4:**
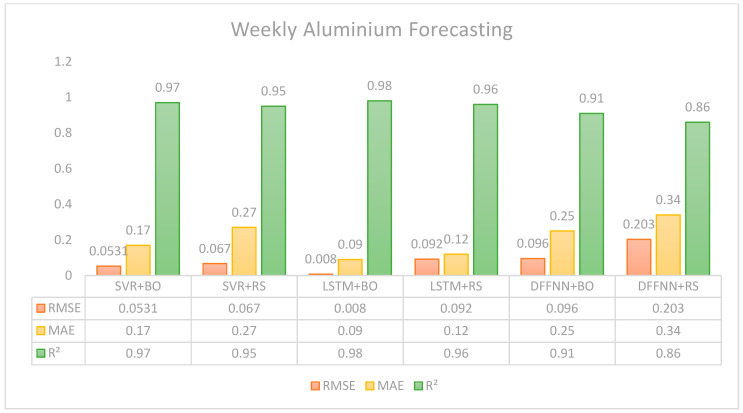
Forecasting results based on weekly data.

Regarding the weekly forecasting task, as shown in Figure 4, one can, in general, observe lower statistical values. Even though some configurations are better than others, the three models with the two hyperparameters present valuable results. Among the models, LSTM with Bayesian optimization demonstrated superior accuracy, achieving an RMSE of 0.008, MAE of 0.09, and an R^2^ of 0.98, indicating that it could explain 98% of the variance in weekly aluminum prices. This is, in fact, a very good performance for the time frame. This model significantly outperformed the other configurations, illustrating its robustness in capturing the temporal dynamics critical for accurate weekly predictions. Conversely, the DFFNN model, particularly when tuned with random search, displayed the weakest performance, with the highest RMSE and MAE values and the lowest R^2^, suggesting considerable prediction inaccuracies. The SVR model showed moderate performance, with Bayesian optimization providing markedly better results than random search.

In summary, Figure 4 shows that the LSTM optimized via Bayesian optimization emerges as the best-performing model on weekly data. This performance indicates that the LSTM model not only predicts weekly aluminum prices with minimal error but also explains the variance in the data at a very high percentage, showcasing its excellent fit and predictive reliability for this sample rate. Referring to the graph presented, it is important to note that the best-performing model for random search is also LSTM. Such consistent performance across different hyperparameter tuning methods highlights LSTM’s robust adaptability and effectiveness in handling the daily and weekly spot prices of aluminum and demonstrates that it is particularly well-suited to the characteristics inherent in the dataset.

**Figure 5:**
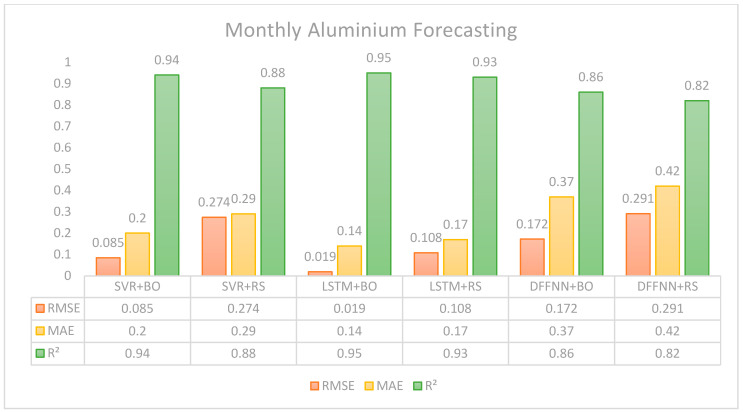
Forecasting results based on monthly data.

Finally, with respect to the monthly price forecasting task, Figure 5 shows that the SVR performs significantly better under Bayesian optimization, showing a much lower RMSE of 0.085 against 0.274 RMSE of random search and 0.2 MAE, slightly better than 0.29 from random search. The difference relies on the accuracy variability of the model, where a higher 0.94 R^2^ is identified against a 0.88 R^2^ of random search. LSTM with Bayesian optimization shows a low 0.019 RMSE and 0.14 MAE, along with the highest 0.95 R^2^ from the rest of the models, demonstrating prediction accuracy and model fit. It outperforms the random search configuration, but even under random search, LSTM still presents good performing results with a 0.108 RMSE, 0.17 MAE, and a very good 0.93 R^2,^ which means the model can explain the variability of the data. The DFFNN model shows the weakest performance among the others, particularly with random search, which yields the highest RMSE (0.291) and MAE (0.42), and the lowest R^2^ (0.82). The performance is slightly improved with Bayesian optimization, but it still underperforms the other models.

In summary, according to Figure 5, the ability of LSTM to handle temporal dependencies and non-linear relationships is particularly advantageous even for longer-term forecasting horizons, for instance, monthly data. For this study, we can observe how the R^2^ values remain high among all models for all time horizons under LSTM. Even though the DFFNN model did not perform as well as the LSTM, we can still conclude that DFFNN shows high performance in forecasting aluminum spot prices under all three horizons.

## 4. Discussion and Conclusions

Time series forecasting is a challenging task in various problems, including meteorology [28], finance [29,30,31,32], public health [33], supply chain management [34], air pollution monitoring [35,36], energy markets [37,38,39,40], rolling bearing life prediction [41], and macroeconomic indicators prediction [42].

The current role of aluminum is increasing its interest worldwide. Indeed, due to its diverse qualities, cost efficiency, and sustainability, aluminum is a prevalent non-ferrous metal used in several industries. In this study, the historical spot prices of aluminum from the LME index were analyzed over the period from 16 June 2014 to 22 February 2024. These were used to predict pricing trends into daily, weekly, and monthly aluminum price forecasts. By comparing deep learning models (LSTM, DFFNN) and SVR alongside two hyperparameter optimizers (Bayesian optimization and random search), this study aims to develop a simple, accurate, and time-efficient forecasting model tailored for supply chain practitioners. Recently, BO was successfully applied in optimization of machine learning with applications to different problems [43,44,45,46,47,48]. Similarly, RS was found to be effective in machine learning applications, including unsupervised clustering [49] and feature selection [50].

The experimental results show evidence that the trained LSTM-BO emerges as the best-performing predictive system across the three time horizons, for instance, daily, weekly, and monthly sampling. By consistently achieving the lowest RMSE, MAE, and highest R^2^, the LSTM-BO surpassed the other implemented models, including the SVR-BO, DFFNN-BO, LSTM-RS, SVR-RS, and DFFNN-RS. Notably, models utilizing Bayesian optimization consistently outperform those using random search in each sample rate. The results indicate that the LSTM model consistently outperforms other models across different hyperparameter tuning methods, underscoring its robustness and suitability for volatile datasets such as aluminum spot prices. This consistent performance, observed with both Bayesian and random search optimization algorithms, confirms that LSTM is an effective model to be considered when dealing with aluminum markets. Indeed, the results of the best-performing model, LSTM-BO, demonstrate the strength of the Bayesian optimization method and LSTM to achieve accurate forecasting compared to reference models, including DFFNN and SVR.

Additionally, we performed three statistical tests to check differences between the best model, namely the LSTM when tuned by BO and RS, and the optimized LSTM against true observations. Specifically, we applied Student’s *t*-test for the null hypothesis of means, *F*-test for the null hypothesis of equality of variances, and Kolmogorov–Smirnov test for the null hypothesis of similar distributions at 5% statistical significance level. The computed *p*-values (probability values) from statistical tests are provided in Table 1. As shown, the null hypothesis of equality of means and variances between LSTM-BO and LSTM-RS is accepted under daily, weekly, and monthly sampling. Similarly, the null hypothesis for the distributions of forecasts from LSTM-BO and LSTM-RS is accepted. Therefore, their forecasts are statistically similar. Also, the null hypothesis of equality of means and variances between LSTM-BO and true observations is accepted under daily, weekly, and monthly sampling. Likewise, the null hypothesis for the distributions of forecasts from LSTM-BO and true observations is accepted. Furthermore, the null hypothesis of equality of means and variances between LSTM-RS and true observations is accepted under daily, weekly, and monthly sampling. In the same way, the null hypothesis for the distributions of forecasts from LSTM-RS and true observations is accepted.

Moreover, to determine if the two best predictive models (LSTM-BO and LSTM-RS) have significantly different predictive accuracy, the standard Diebold–Mariano (DM) test is performed. The DM statistic and *p*-value of the test are provided in Table 2 with respect to the null hypothesis that their expected losses are the same. As shown, the predictive accuracies of the LSTM-BO and LSTM-RS are statistically different at daily and weekly horizons. On the contrary, the predictive accuracies of the LSTM-BO and LSTM-RS are not statistically different at monthly horizons.

Finally, the time complexity of each model follows. The time complexity of the LSTM is *O*(*l*·*h*·(*d* + *h*)), where *l* is the sequence length, *d* is the input dimension, and *h* is the hidden state dimension. For the multilayer deep neural network, the time complexity is *O*(*l*·*p*) where *p* is the total number of parameters. Lastly, the time complexity of the SVR is *O*(*l*^3^·*k*) where *k* is the number of support vectors.

In this study, unlike previous works [1,8,10,12], we did not rely on data decomposition, as many techniques, including signal processing and adaptive signal processing, require appropriate selection of analytical functions, decomposition level, and stopping values, to name a few. Such an investigation is out of the scope of the current work. Indeed, we rely on historical data to make the model simple to implement, understand, and interpret based on past observations. In addition, contrary to previous works [1,4,5,6,7,8,9,10,11,12], we examined the effect of time horizon (for instance, daily, weekly, monthly) on the performance of the predictive models to draw general conclusions. Furthermore, to enrich the existing literature, we examined the type of optimization on the performance of the predictive models. Particularly, we compared the effect of two optimization techniques to tune the models, for instance, BO versus RS. In summary, compared to the existing literature, in our study, the implemented models are expected to be simple, fine-tuned and effective, and generalized to different time horizons.

The implications of the main findings are as follows. The LSTM model with Bayesian optimization demonstrated a great performance throughout the weekly sample rate, yielding highly reliable results. Applying this model to weekly forecasting of aluminum prices can significantly enhance supply chain management. It allows procurement managers to refine their sourcing strategies, optimize their planning, and develop robust purchasing plans contributing to sustainable inventory management. This approach promises more strategic insights and improved decision-making for supply chain operations. One could think that with a weekly time horizon, the model will drastically increase error metrics, mainly because it is being trained with fewer observations to learn from. However, the learning ability of the LSTM compensates for the fewer data points by improving stability, helping the model to capture patterns and improve performance. Monthly forecasts, on the other hand, provide a broad overview useful for strategic planning in supply chain management, but they come with limitations. For instance, monthly forecasts might not encompass a series of influences such as macroeconomic changes, which may be important to consider and are not included in short-term forecasts. Thus, while monthly forecasts offer valuable insights into long-term planning, they must be approached with caution to mitigate the potential for loss of detailed information, which can lead to a higher error rate.

While SVR is a robust statistical learning model, it has been surpassed by neural networks that excel in learning from data dependencies, such as LSTM. Additionally, the DFFNN model was adapted into a deep neural network by increasing its complexity to four layers to better handle non-linear dynamics in aluminum spot prices. However, despite these adjustments, it still fell short of LSTM’s performance. The enhanced configuration of LSTM not only outperformed DFFNN, which showed the lowest performance metrics, but also demonstrated its superior ability to manage complex, volatile datasets effectively.

Throughout this study, the random search algorithm has proven to be a competent hyperparameter tuning method, yielding good results though not as efficient as those achieved by Bayesian optimization. While Bayesian hyperparameters enhance accuracy due to their systematic approach to tuning, random search offers simplicity and reduced computational effort. This makes random search particularly valuable in scenarios with limited computational resources, where its ease of implementation and efficiency may justify the slight trade-off in performance metrics.

For comparison with previous works that used the same data source [27], the VMD-LSTM model achieved an RMSE of 2.83 and MAE of 2.01 [3]; the predictive model based on Prophet improved complete ensemble empirical mode decomposition with adaptive noise, and multi-model error correction achieved an RMSE of 0.37 and MAE of 0.63 [7]; the model based on complete ensemble empirical mode decomposition with adaptive noise, VMD, PSO, and LSTM obtained an RMSE of 1.88 and MAE of 4.21 [8]; and LSTM-GRU yielded to an RMSE of 31.84, MAE of 21.73, and R^2^ of 0.99 [11]. Additionally, our proposed LSTM-BO trained with past observations achieved an RMSE of 0.004, MAE of 0.08, and R^2^ of 0.99. Therefore, our proposed predictive model of aluminum price is simple to implement and accurate compared to the literature. However, this comparison should be made with caution as the sample period is different.

In summary, we conclude that the LSTM-BO is the best predictive system across all time horizons (daily, weekly, and monthly). Indeed, the LSTM-BO is highly beneficial for aluminum spot price forecasting. In addition, the Bayesian optimization improves the accuracy of the predictive systems compared to the random search.

This study has potential limitations. First, the analysis relies exclusively on historical aluminum price data and does not incorporate exogenous variables such as macroeconomic indicators, energy prices, and inventory levels. This can be explained by the fact that several macroeconomic variables are not recorded on a daily or weekly basis. In addition, inventory levels are not available at the company, national, and international levels. Second, since the LSTM-BO model is basically an artificial neural network, it is difficult to explain the contribution of each input variable in price forecasting.

Lastly, future work would consider incorporating economic and financial data as covariates to improve prediction, especially for monthly forecasts. Additionally, applying feature selection methods would be applied to select the best covariates, as previous works showed that feature selection helps improve the accuracy of predictive systems [44,51,52,53,54,55,56,57,58,59,60,61,62]. In addition, feature selection could be useful to explain the performance of LSTM-BO. Another future research direction is considering explainable artificial intelligence (xAI) to explain the performance of the AI-based predictive system. Also, as decomposition helps extract meaningful traits for prediction purposes with applications in economics and finance [63,64,65,66,67,68] and engineering [69,70,71,72,73,74,75,76], future work may involve the use of data-adaptive decomposition methods to enhance the practicality and adaptability of the proposed predictive models.

## Figures and Tables

**Table 1:** Reported *p*-values from standard two-sample statistical tests.

	LSTM-RS Versus LSTM-BO	LSTM-RS Versus Actuals	LSTM-BO Versus Actuals
	*p*-Value	*p*-Value	*p*-Value
**Daily forecasts**			
Student’s *t*-test	0.7328	0.6816	0.9455
*F*-test	0.9518	0.8884	0.9518
Kolmogorov–Smirnov test	0.89	0.7503	1
**Weekly forecasts**			
Student’s *t*-test	0.7749		
*F*-test	0.8998	0.9071	0.8998
Kolmogorov–Smirnov test	0.6022	0.9367	0.6022
**Monthly forecasts**			
Student’s *t*-test	0.9808	0.668	0.9808
*F*-test	0.8713	0.913	0.8713
Kolmogorov–Smirnov test	0.9983	0.1359	0.1359

The Student’s *t*-test is used to test the null hypothesis that the forecasts from LSTM-RS and LSTM-BO have equal means. The *F*-test is used to test the null hypothesis that the forecasts from LSTM-RS and LSTM-BO have equal variances. The Kolmogorov–Smirnov test is used to test the null hypothesis that the forecasts from LSTM-RS and LSTM-BO have similar distributions. All statistical tests are performed at 5% significance level.

**Table 2:** Reported *p*-values from Diebold–Mariano test to check differences between best models: LSTM-RS and LSTM-BO.

	D-M Statistic	*p*-Value
Daily	9.9183	3 × 10^−21^
Weekly	2.4985	0.0143
Monthly	0.5661	0.5803

The Diebold-Mariano (D-M) test is applied to loss functions from the best models, namely the LSTM-BO and LSTM-RS, to determine if they have significantly different predictive accuracy. Specifically, it tests the null hypothesis that the expected losses of LSTM-BO and LSTM-RS are the same. The test is performed at 5% statistical significance level.

## Data Availability

Data source is provided in reference [27].

## Abbreviations

The following abbreviations are used in this manuscript:

LSTM	Long short-term memory networks
DFFNN	Deep feedforward neural networks
BO	Bayesian optimization
RS	Random search
SVR	Support vector regression
RMSE	Root mean squared errors
MAE	Mean absolute errors
R^2^	Coefficient of determination, R-squared.
